# Preliminary study for developing a fully biological breast implant

**DOI:** 10.1016/j.jpra.2025.10.012

**Published:** 2025-10-16

**Authors:** Victor Pozzo, Laurent A. Lantieri

**Affiliations:** Service de Chirurgie Plastique, Hôpital Européen Georges Pompidou, Assistance Publique-Hôpitaux de Paris (APHP), Université Paris Descartes, Paris, France

**Keywords:** Breast reconstruction, Innovation, Biological implants, Research

## Abstract

**Background:**

Breast cancer reconstruction remains limited by complications associated with current techniques. This study evaluates a novel biological breast implant composed of an acellular dermal matrix (ADM) shaped like a silicone implant, combined with natural biological content, including autologous fat, in a single porcine model.

**Methods:**

Four implant types were tested in subcutaneous pockets of a Yorkshire pig: implants filled with autologous fat and dermal microbeads, implants filled with autologous fat alone, implants filled with dermal microbeads alone, and an implant made entirely of P4HB mesh. Clinical, physical, and histological evaluations were performed over three months, focusing on inflammation, neovascularization, and tissue integration.

**Results:**

The implant filled with autologous fat and dermal microbeads demonstrated good integration, moderate neovascularization, and fibrous encapsulation, though some steatonecrosis occurred. The implant filled with autologous fat alone failed structurally by day 60. Implants filled with dermal microbeads and the P4HB mesh implant exhibited poor integration and rigidity.

**Conclusion:**

This study represents a first step in developing a fully biological breast implant. While the combination of an ADM implant with natural fillers such as autologous fat and dermal microbeads shows promise, further experiments are necessary to refine the implant's content. Future research will focus on optimizing the ratio of fat to microbeads and exploring alternative fillers, such as hyaluronic acid or collagen gel, to improve adipocyte viability, vascularization, and long-term functionality.

## Introduction

Breast cancer remains the most common cancer in women across developed countries, with 60,000 cases and 20,000 mastectomies annually in France. However, only 40 % of patients undergo reconstruction, often due to limited access, insufficient information, or reluctance to face further procedures. Current reconstruction methods, including silicone implants and autologous tissue transfer, have significant limitations. Silicone implants present risks such as infections, capsular contracture, and the need for revision surgeries,^1^^,^^2^ while autologous reconstruction causes donor-site morbidity and scarring.^3^

Recent advancements in acellular dermal matrices (ADMs) derived from porcine sources have shown promise in enhancing implant stability and tissue integration.^4^ This study explores a next-generation biological breast implant, combining ADMs with autologous fat in an animal model. The aim is to assess implant feasibility and safety over three months, focusing on adipocyte viability and tissue integration. If successful, this approach could integrate the benefits of implants and autologous reconstruction while reducing complications.

## Materials and methods

### Animal model and ethical approval

A 70-kg Yorkshire pig was selected due to its anatomical and physiological similarities to humans, particularly in adipose tissue characteristics. The study protocol received approval from the French Ministry of Research and Higher Education (APAFIS #39499–2022052311138984 v4). All procedures adhered to ethical standards for animal research.

### Implant design and composition

The implants, designed by Meccelis Biotech® (La Rochelle, France), mimicked the shape of a conventional breast implants with a 150-cc capacity. They consisted of a shell made from decellularized porcine dermis reinforced with poly-4-hydroxybutyrate (P4HB), a biodegradable polymer commonly used in reconstructive surgery which enhance mechanical stability. This shell was filled with various components as follows: Implant 1 contained 100 mL of autologous fat combined with 50 mL of decellularized dermal microbeads. Implant 2 was filled with 150 mL of autologous fat. Implant 3 contained 150 mL of decellularized dermal microbeads. Implant 4 consisted solely of a mesh structure made from P4HB.

### Surgical procedure

Under anesthesia, liposuction was performed to harvest 1000 mL of fat from the flanks, abdomen, and neck. The fat was decanted for 15 min before use. Four subcutaneous pockets were created at the junction of the flanks and iliac fossae, with two pockets on each side. Implants were inserted, and incisions were closed using Monocryl 2/0 sutures with intradermal running stitches. Surgical glue was applied for reinforcement. Postoperative care included a 48-hour antibiotic prophylaxis with intramuscular Vetrimoxin.

After three months, implants were explanted under general anesthesia.

### Follow-up and evaluation

The implants were monitored over three months using clinical, physical, and histological assessments.

• Clinical: The animal was observed for signs of infection, inflammation, or adverse reactions.
• Physical: Implant characteristics, including softness, shape retention, and texture changes, were recorded periodically.
• Histological: Tissue samples were fixed in formalin, stained with hematoxylin and eosin (H&E), and analyzed for inflammation, necrosis, neovascularization, scaffold degradation, and cellular colonization following ISO 10993 standards.

## Results

### Clinical outcomes

The implants were well tolerated without signs of systemic infection. However, Implant 2 (fat alone) failed on Postoperative Day (POD) 60, developing a large cyst of cytosteatonecrosis that led to implant exposure. Despite this, no severe adverse reactions occurred.

### Physical characteristics

Implant 1, composed of fat and dermal microbeads, retained its projection over the three-month period but exhibited some loss of softness. Implant 2, containing only fat, initially increased in size but failed structurally by postoperative day 60. Implants 3 and 4, made of dermal microbeads alone and a P4HB mesh respectively, both became rigid and lost their projection within just 15 days.

### Histological analysis

Implant 1 exhibited moderate neovascularization and fibrous encapsulation, with good tissue integration. Most of the adipose tissue was preserved, although some areas of steatonecrosis were present. Fibroblast infiltration suggested active tissue remodeling. Implant 2 had no available histological data due to its failure. Implant 3 showed significant inflammatory infiltration with lymphocytes and eosinophils but lacked neovascularization. Implant 4 demonstrated sparse neovascularization and thick fibrous encapsulation, indicating poor integration.

## Discussion

This study highlights the potential of decellularized porcine dermis as a scaffold for biological breast implants and underscores the importance of implant content in maintaining viability and function. While the scaffold provided structural integrity and supported neovascularization, implant failure due to necrosis^5^ emphasizes the need for optimized fillers. Fat alone proved insufficient because of poor vascular integration, leading to necrosis and volume loss. However, the combination of fat with dermal microbeads showed promise, suggesting that integrating biological components can improve tissue survival and vascularization. This paper presents a proof-of-concept study designed to demonstrate the feasibility of our approach and to lay the groundwork for future research. We fully acknowledge its limitations, including the absence of a control group, the relatively short study duration (3 months), the evaluation of a single volume (150 mL), the lack of objective mechanical assessments, and the limited sample size. Future research should focus on optimizing implant content to enhance adipocyte survival. One key strategy involves refining the ratio of fat to dermal microbeads to improve long-term stability. Another approach is to explore alternative fillers such as collagen gels, which could support adipocyte colonization. Additionally, incorporating oxygen transport molecules like Hemo2life® may enhance cellular viability.

**Figure 1:**
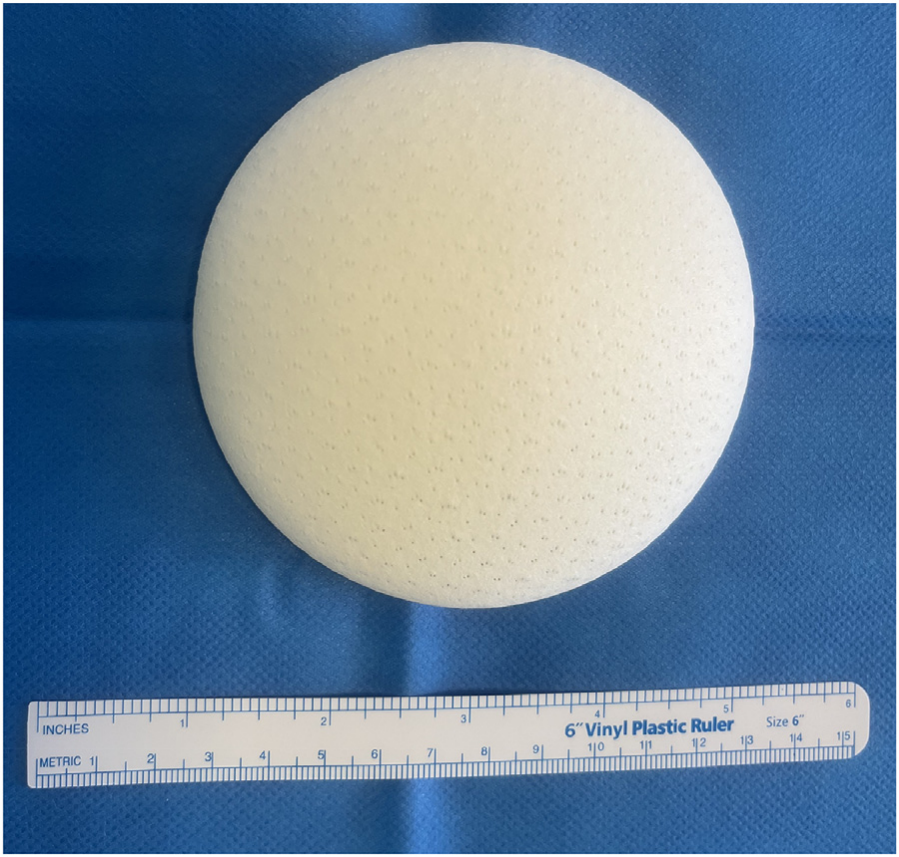
Biological implant. (a) Visual of the dry biological implant composed of P4HB and decellularized porcine dermis.

**Figure 2:**
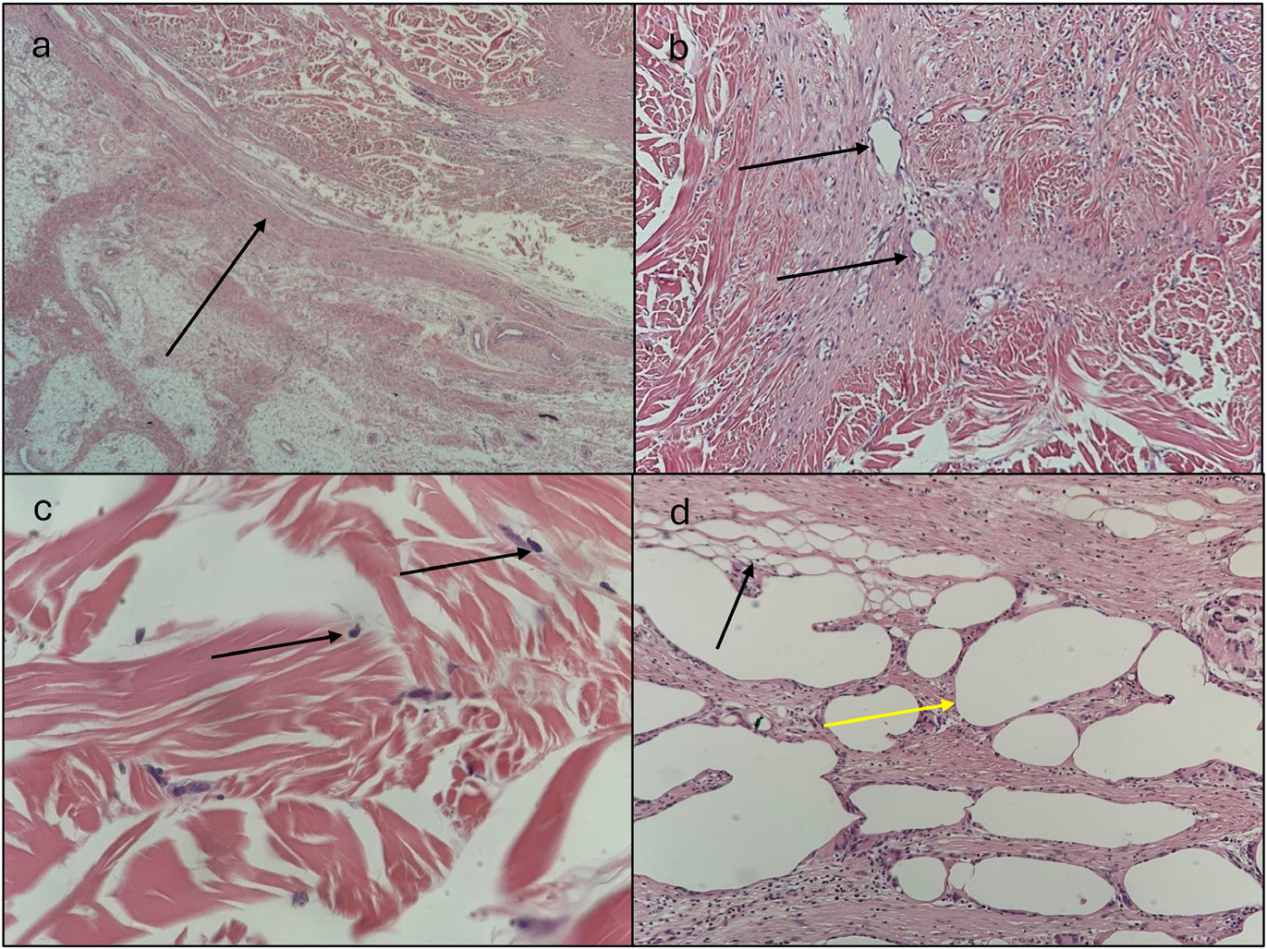
Histology (H&E) of implant 1 (fat + dermal microbeads). (a) Encapsulation of the implant showing with the arrow a moderately thick fibrous band, indicating good integration with the surrounding tissue (H&E, 200x magnification); (b) Neovascularization within the implant, with the presence (arrows) of newly formed blood vessels (H&E, 100x magnification); (c) Fibroblast proliferation within the implant, with black arrows showing visible fibroblast nuclei colonizing the scaffold (H&E, 400x magnification); (d) Fat within the implant with yellow arrows showing areas of cytosteatonecrosis alongside preserved adipose tissue showed with black arrows (H&E, 100x magnification).

To validate these approaches, the next phase of research will involve both in vitro and in vivo studies, using MRI to monitor implant evolution over time. These efforts aim to develop a clinically viable, fully biological breast implant that combines aesthetic and functional benefits while minimizing complications (Figures 1 and 2).

## Funding

This study was fully funded by Meccellis Biotech®. The company was not involved in the writing of this manuscript or in the decision to submit it for publication.

## Ethical statement

The study protocol received approval from the French Ministry of Research and Higher Education (APAFIS #39499–2022052311138984 v4). All procedures adhered to ethical standards for animal research.

## Declaration of competing interest

V. Pozzo and L. Lantieri declare no conflicts of interest.
